# Agreement between the International Physical Activity Questionnaire and Accelerometry in Adults with Orthopaedic Injury

**DOI:** 10.3390/ijerph17176139

**Published:** 2020-08-24

**Authors:** William G. Veitch, Rachel E. Climie, Belinda J. Gabbe, David W. Dunstan, Neville Owen, Christina L. Ekegren

**Affiliations:** 1 Department of Epidemiology and Preventive Medicine, Monash University, Melbourne 3004, Australia; bill.veitch@monash.edu (W.G.V.); belinda.gabbe@monash.edu (B.J.G.); 2 Sports Cardiology, Baker Heart and Diabetes Institute, Melbourne 3004, Australia; rachel.climie@baker.edu.au; 3 Health Data Research UK, Swansea University Medical School, Swansea SA2 8QA, UK; 4 Physical Activity Laboratory, Baker Heart and Diabetes Institute, Melbourne 3004, Australia; David.Dunstan@baker.edu.au; 5 Behavioural Epidemiology, Baker Heart and Diabetes Institute, Melbourne 3004, Australia; Neville.Owen@baker.edu.au; 6 Swinburne Centre for Urban Transitions, Swinburne University of Technology, Melbourne 3122, Australia; 7 Emergency and Trauma Centre, The Alfred, Melbourne 3004, Australia; 8 Rehabilitation, Ageing and Independent Living (RAIL) Research Centre, Monash University, Frankston 3199, Australia

**Keywords:** sitting, sedentary behaviour, IPAQ, validation, accelerometer, activity

## Abstract

Orthopaedic injury can lead to decreased physical activity. Valid measures for assessing physical activity are therefore needed in this population. The aim of this study was to determine the agreement and concordance between the International Physical Activity Questionnaire–Short Form (IPAQ) and device-measured physical activity and sitting time in orthopaedic injury patients. Adults with isolated upper or lower limb fracture (*n* = 46; mean age of 40.5 years) wore two activity monitors (ActiGraph wGT3X-BT and activPAL) for 10 days, from 2 weeks post-discharge. The IPAQ was also completed for a concurrent 7-day period. Lin’s concordance correlation coefficients and Bland–Altman plots were calculated to compare walking/stepping time, total METmins, and sitting time. The IPAQ overestimated device-derived walking time (mean difference = 2.34 ± 7.33 h/week) and total METmins (mean difference = 767 ± 1659 METmins/week) and underestimated sitting time (mean difference = −2.26 ± 3.87 h/day). There was fair concordance between IPAQ-reported and device-measured walking (ρ = 0.34) and sitting time (ρ = 0.38) and moderate concordance between IPAQ-reported and device-measured METmins (ρ = 0.43). In patients with orthopaedic injury, the IPAQ overestimates physical activity and underestimates sitting time. Higher agreement was observed in the forms of activity (walking, total PA and sitting) commonly performed by this patient group.

## 1. Introduction

Globally in 2013, approximately 21.7 million people sustained a fracture that received inpatient care [1], with younger men and older women being most at risk [2]. Low levels of physical activity and high levels of time spent sitting have been observed in people who have sustained an orthopaedic injury up to 18 months post-injury [3]. Thus, orthopaedic injury has the potential to be a catalyst for developing subsequent chronic diseases associated with prolonged inactivity and sedentary behaviour [4].

Physical inactivity—insufficient levels of physical activity (any bodily movement produced by skeletal muscles that results in energy expenditure) [5]—is a worldwide public health concern and is associated with an increased risk of obesity, type 2 diabetes, heart disease and cancer [6]. Further, more than 4 h of sedentary behaviour (any waking behaviour characterized by energy expenditure ≤1.5 metabolic equivalents [METs], while in a sitting, reclining or lying posture) [7] per day can be associated with an increased risk of chronic disease [4], with each additional hour of sitting increasing the odds of multimorbidity [8].

Valid self-reported measures are a vital component in improving our understanding of physical inactivity and sedentary behaviour both before and after injury. The International Physical Activity Questionnaire-Short Form (IPAQ) has been widely used as a self-reported measure of physical activity and sitting time. The IPAQ’s validity has been assessed in a range of clinical [9,10,11,12,13] and healthy [12] populations. However, the IPAQ has not been validated against device-based measures in people with injuries. The unique characteristics of people with injuries, such as the sudden onset of physical activity limitations and a self-reported pre-injury health status higher than the population norm [14], highlight a need to ensure the validity of self-reported measures against their objective alternatives in this context [15]. In doing so, this may enable the widespread utilisation of these questionnaires in assessing changes to physical activity behaviour and the development of tailored interventions for patients who would have otherwise been without support.

Accordingly, the aim of this study was to determine the agreement and concordance between the self-reported Short-Form IPAQ and device-measured physical activity and sitting time in people with orthopaedic injuries.

## 2. Materials and Methods

Participants were recruited as inpatients receiving treatment for an isolated (non-pathologic) upper limb or lower limb fracture in a major (Level 1) trauma centre in Victoria, Australia. Patients were excluded if they had a severe traumatic brain injury, a spinal cord injury, pre-existing cognitive deficits or spoke a language other than English. Ethical approval was obtained from the Monash University and The Alfred Hospital (458/16) human research ethics committees. All participants provided written informed consent to participate.

This study was completed at the largest trauma centre in Australia, meaning that it is likely that the captured sample is representative of orthopaedic trauma patients within the 18 to 69 age range. However, the Alfred Hospital is situated in the inner metropolitan area, so it is possible that a greater number of certain causes of injury, such as cycling accidents, may have been included in the study sample. It is also possible that those individuals opting to participate in the study had an interest in physical activity, potentially more so than the general population.

Each participant was provided with two previously validated activity measurement devices, the ActiGraph wGT3X-BT triaxial accelerometer (ActiGraph LLC, Pensacola, FL, USA) [16] and the activPAL accelerometer/inclinometer (PAL Technologies Ltd. Glasgow, UK) [17,18], two weeks after hospital discharge. At this time, participants were shown how the devices worked, how to fit them and instructed to wear both devices for the subsequent 10-day period. The ActivPAL was worn on the anterior aspect of the upper leg and was worn 24 h a day underneath a waterproof adhesive dressing. The Actigraph was worn around the waist and worn during waking hours, other than when showering/swimming. Date of birth, sex, self-reported height and weight, and date of injury were also obtained. An activity diary, adapted from a previous study [19], was provided to participants in order to refine non-wear/sleep periods during the 10-day monitoring period. On the final day of monitoring, each participant was contacted by telephone to complete the IPAQ relating to the most recent seven consecutive days coinciding with device-based monitoring (Figure S1).

The IPAQ-Short Form consists of seven questions that assess the amount of time spent sitting, walking and participating in moderate- and vigorous-intensity physical activity (MPA and VPA, respectively) [20]. As outlined in the IPAQ Group’s guidelines for data processing [20], self-reported responses where the total of all walking, MPA and VPA time was more than 16 h, were truncated to account for an expected 8 h per day for sleeping. The guidelines also specify that events shorter than 10 min should be recoded as zero and, therefore, data from both self-reported and device-based methods were removed when the bout lasted less than 10 continuous minutes. Time spent in each type of physical activity was also truncated to a maximum of three hours per day, another stipulation of the guidelines [20]. No alterations were made to sitting-time data.

The ActiGraph was used to determine time spent performing MPA and VPA and data were sampled at 30 Hz for the 10-day monitoring period. The activPAL was used to determine the total daily amount of time spent stepping and sitting [18]. Both devices have been previously validated in general and slow-walking populations [18], but not specifically in an orthopaedic injury population. The activPAL was worn on the unaffected lower limb if the patient was non-weightbearing. All data were extracted using the proprietary software for each device (activPAL: activPAL™ professional (Version 7.2.32), ActiGraph: ActiLife (Version 6.13.3). Activity data from both devices were processed in SAS (version 9.3, SAS Institute, Cary, NC, USA). Non-wear periods were determined for ActiGraph data using the Choi algorithm [21]. Once sleep periods had been removed from activPAL 24-h data, the algorithm outlined by Winkler et al. [19] was used to determine activPAL valid days. To account for the fact that orthopaedic injury patients are likely to be less active than the general population [22,23], the “any one activity that accounts for >95% of waking wear time” condition [19] was removed and the step threshold [19] was lowered from 500 to 100 steps per day. For both devices, all days where participants reported less than 600 min of waking wear time were ruled invalid. To be included in analyses, participants were required to complete at least four valid days of device-based monitoring within the 7-day self-reported timeframe.

Freedson cut-points [24], to determine moderate and vigorous physical activity time, were applied to ActiGraph data. Total daily physical activity (METmins) was calculated by first multiplying the time spent performing a particular intensity level of activity by a pre-determined weighting factor (Walking: 3.3 METs, MPA: 4.0 METs, VPA: 8.0 METs) [25]. Then, the calculated METmins for each variable were combined to produce the total physical activity value [20]. Stepping time was calculated by subtracting the total time spent performing MPA and VPA (as determined by the ActiGraph) from the total stepping time (as determined by the activPAL). This method was utilised in order to ensure the use of data from the most valid device for each intensity level. The walking data recorded by the ActivPAL are considered to be more representative of walking at a pace at or below normal daily activity [18] and the ActiGraph is considered to provide a more valid representation of both moderate- and vigorous-intensity physical activity [26,27]. While it must be acknowledged that this combination of measures allows for a potential reduction in device-based walking, this method of recording activity in a step-down and mutually exclusive fashion is consistent to the IPAQ [20].

Bland–Altman plots were used to determine the agreement between self-reported and device-based measures of mean seven-day walking/stepping time, total seven-day METmins of physical activity, and mean daily sitting time. Because all three variables were positively skewed, a trend was applied to the Bland–Altman plots [28]. Applying this trend allowed for a representation of the change in average agreement and the variability in agreement with changes in values. The average mean difference between measures for each variable was calculated as the mean difference between the two measures at the average of IPAQ and device-derived values. This was the value used to represent average over/underestimation. In order for a participant to be included in the Bland–Altman analysis for physical activity, a complete dataset for both monitors was required, while, for sitting time, a complete activPAL dataset was required.

Lin’s Concordance Correlation Coefficient (LCCC) [29] was used to assess concordance between self-reported and device-based measures of walking time, MPA, VPA, METmins and sitting time [30]. LCCC was used because, unlike previously utilised methods (Intraclass Correlation Coefficient [31], Pearson’s Correlation Coefficient [11] and Spearman’s Correlation Coefficient [13]), LCCC have been shown to provide a more accurate assessment of agreement due to the fact that they take bias into account [30]. The cut-points outlined by Landis and Koch [32] were used to classify LCCCs, with 0 to 0.20 representing slight concordance; 0.21 to 0.40 fair; 0.41 to 0.60 moderate; 0.61 to 0.80 substantial; and 0.81 to 1.00 almost perfect.

In accordance with the IPAQ scoring protocol, both self-reported and device-measured physical activity data were categorised as low, medium and high. The algorithm used to define these groups takes into account both the overall quantity as well as the frequency of MPA, VPA and walking [20]. Cohen’s kappa was used to determine the agreement between categories of self-reported and device-based physical activity and a weighting was applied to differentiate disagreements of differing seriousness [33] (p. 892). Weighted Kappa values were calculated using the system-defined weights in Stata [34] and the bootstrapping method with 1000 repetitions was used to determine 95% confidence intervals. The following scale was used to determine the level of agreement between self-reported and device-based measures: 0.01–0.20 slight agreement; 0.21–0.40 fair; 0.41–0.60 moderate; 0.61–0.80 substantial; 0.81–1.00 almost perfect [32]. All data were analysed in Stata 15 (StataCorp LLC. College Station, TX, USA) and significance was set at *p* < 0.05.

## 3. Results

**Figure 1:**
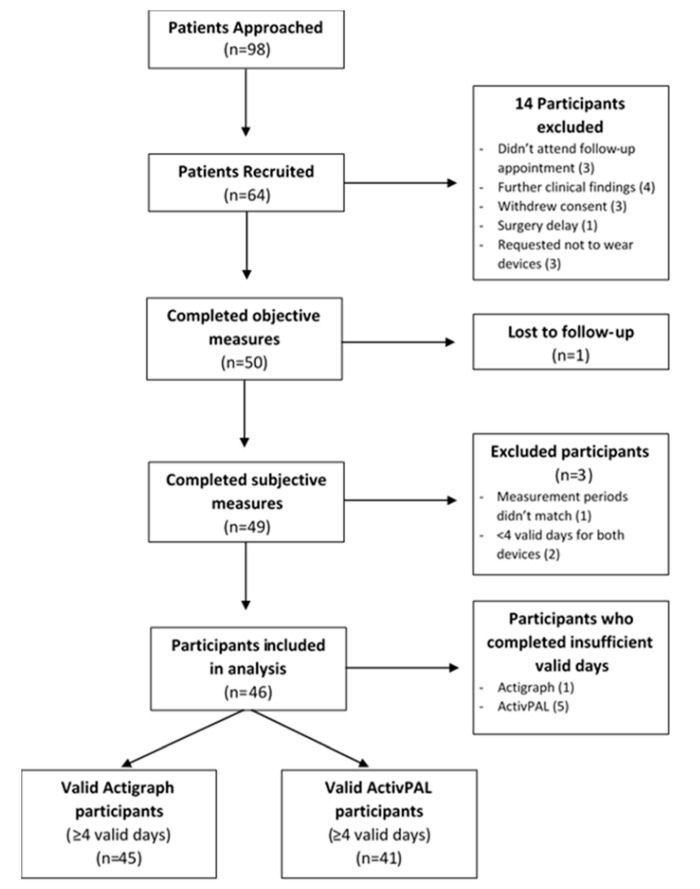
Patient recruitment flowchart.

A total of 64 participants were initially recruited; 17 were excluded for various reasons. Additionally, a further one participant completed insufficient ActiGraph wear days and five participants completed insufficient activPAL wear days. From the 46 remaining participants, the total number of participants with valid ActiGraph data was 45 and 41 with valid activPAL data (Figure 1).

Participants were predominantly male (*n* = 29), with a mean age of 40.5 years and were of normal weight to overweight (based on BMI). Most had sustained lower limb (52%) injuries and, of the lower limb injury participants, 83% were non-weightbearing on the affected limb during the monitoring period (Table 1).

### 3.1. Walking

**Figure 2:**
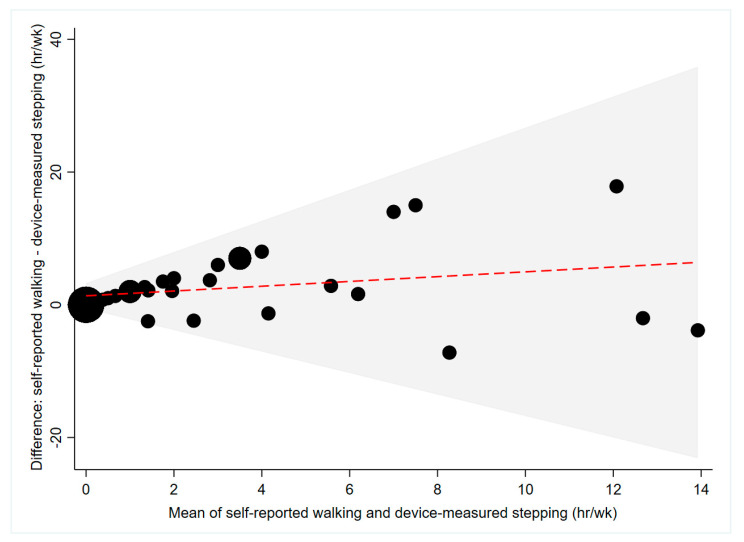
Agreement between self-reported and device-measured walking/stepping time (*n* = 40). The red line represents the mean difference, the grey shaded area represents the limits of agreement and the size of the black dots differs depending on the number of participants that correspond to that value.

Median self-reported walking time was higher than median device-measured stepping time (2.25 vs. 0 h/week; Table 2). An average mean difference of 2.34 h/week was obtained from Bland–Altman analyses, with self-reported walking time consistently higher than device-measured stepping time (Figure 2). The 95% limits of agreement were large (±7.33 h/week; Figure 2), rising considerably with increasing mean of self-reported and device-measured walking/stepping time (Figure 2). People with upper limb injuries over-estimated their walking and stepping time to a lesser extent than people with lower limb injuries (2.00 vs. 2.67 h/day; Table 3). Fair concordance (ρ = 0.34, *p* = 0.01) was observed between self-reported and device-measured walking/stepping time (Table 4).

### 3.2. MPA and VPA

A zero value was recorded for both self-reported and device-measured MPA and VPA in 26 and 40 participants, respectively (Table 2). Median self-reported and device-measured MPA and VPA values were zero mins/week (Table 2). Due to the high numbers of zero values for both MPA and VPA, Bland–Altman plots were not appropriate. Fair concordance (ρ = 0.21, *p* = 0.01) was found between self-reported and device-measured MPA and slight concordance (ρ = 0.03, *p* < 0.001) was found between self-reported and device-measured VPA (Table 4).

### 3.3. Total Physical Activity (METmins)

**Figure 3:**
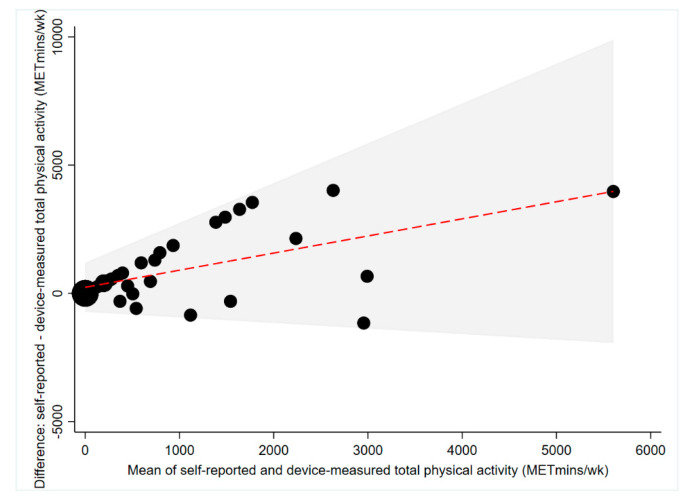
Agreement between self-reported and device-measured total 7-day physical activity (METmins) (*n* = 40). The red line represents the mean difference, the grey shaded area represents the limits of agreement and the size of the black dots differs depending on the number of participants that correspond to that value. *METmins (Metabolic Equivalent minutes).*

Median self-reported total physical activity was higher than median device-measured total physical activity (550 METmins vs. 0 METmins; Table 2). An average mean difference of 767 METmins was observed from Bland–Altman analyses, with self-reported total physical activity consistently higher than device-measured total physical activity (Figure 3). The 95% limits of agreement were large (±1659 METmins; Figure 3), increasing considerably with increasing mean of self-reported and device-measured total physical activity (Figure 3). People with upper limb injuries over-estimated their device-measured total physical activity to a similar extent to people with lower limb injuries (721 METmins/week vs. 760 METmins/week; Table 3). Moderate concordance (ρ = 0.43, *p* < 0.001) was found between self-reported and device-measured total physical activity for the entire sample (Table 4).

### 3.4. Physical Activity Categories

In order to determine the self-reported and objective physical activity category, a complete dataset was required. Therefore, each participant with less than four valid days for either the ActivPAL or Actigraph was excluded, leaving the 40 participants included in the analysis (Table 5). Weighted Kappa analysis showed fair agreement between self-reported and device-measured physical activity categories; ĸ = 0.33 (95%CI: 0.10, 0.57), *p* < 0.01 (Table 5).

### 3.5. Sitting Time

**Figure 4:**
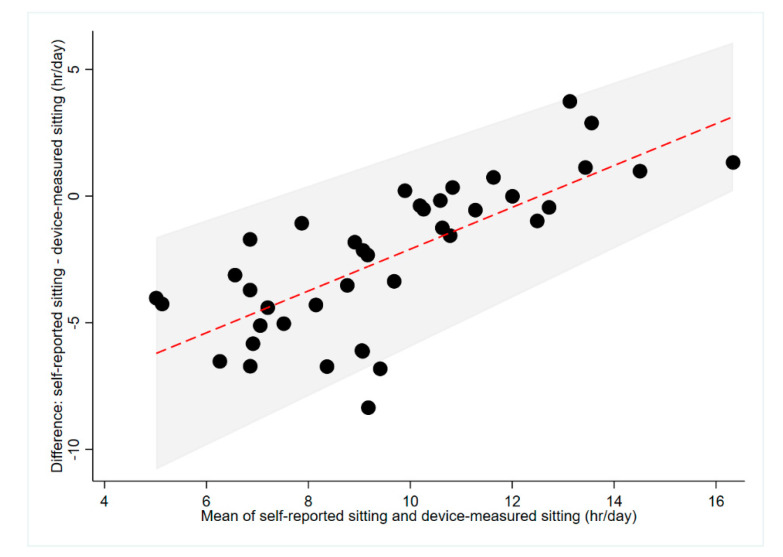
Agreement between self-reported and device-measured sitting time (*n* = 41). The red line represents the mean difference, the grey shaded area represents the limits of agreement and the size of the black dots differs depending on the number of participants that correspond to that value.

Device-measured daily sitting time was higher than median self-reported daily sitting time (10.59 vs. 8.00 h/day; Table 2). An average mean difference of 2.26 h/day was obtained from Bland–Altman analyses, with the majority of participants (<12.47 h/day) underestimating their daily sitting time (Figure 4). Participants who reported sitting for, on average, more than 12.47 h/day were likely to over-estimate their device-measured daily sitting time. The 95% limits of agreement were large (±3.87 h; Figure 4), decreasing minimally with increasing self-reported and device-measured daily sitting time. People with upper limb injuries under-estimated (−3.41 ± 3.34 h/day) their daily sitting time to a greater extent than people with lower limb injury (−1.45 ± 4.48 h/day; Table 3). Fair concordance (ρ = 0.38, *p* < 0.001) was found between self-reported and device-measured daily sitting time for the entire sample (Table 4).

## 4. Discussion

This study is the first to determine the agreement between the self-reported IPAQ and activity monitors for assessing physical activity and sitting time in adults with orthopaedic injuries. The main findings were that participants overestimated device-measured walking time and total METmins and underestimated daily sitting time; there was only fair concordance between IPAQ-reported and device-measured walking and sitting time, and moderate concordance between IPAQ-reported and device-measured METmins; participants with an upper limb injury overestimated walking time to a lesser extent than those with a lower limb injury, and most did not engage in any MPA or VPA. These findings are important because they highlight differences between objective and self-report measures of activity in orthopaedic patients that clinicians should consider when administering a PA questionnaire. Over-estimation of IPAQ-reported MPA, VPA, METmins and total walking time has been reported in most previous research in healthy populations [35,36]. Possible reasons for this over-estimation include over reporting by the participants for social-desirability reasons, or to align their behaviour with their pre-injury activity levels or the recommendations of a healthcare professional. It is also possible that the activity that the participants considered to be walking did not align with what the devices determined as walking, and this warrants further investigation.

In relation to walking time, the concordance between device-based and IPAQ-reported measures used in this study was less than the correlation reported in previous research in healthy (ρ = 0.34 vs. Men: r = 0.56, Women: r = 0.32) [37] and similar to previous research in clinical populations (ρ = 0.34 vs. r = 0.31) [10]. Previous studies [10,37] investigating the level of agreement between the IPAQ and activity monitors for assessing walking time used a hip-mounted accelerometer rather than the thigh-worn activPAL accelerometer utilised in this study. The activPAL has, however, been shown to be a more accurate method for assessing walking time than hip-worn accelerometers, particularly at the slower walking speeds that are likely in this less mobile population [18].

The negligible amounts of moderate and vigorous physical activity reported in this study are similar to previous findings in clinical populations [10], and suggest that measurement of physical activity in people with orthopaedic injury should be predominantly focused on the measurement of walking and light-intensity physical activity (LPA), as these are the most common behaviours in this population. Despite measurement of MPA and VPA alone not being informative in the acute orthopaedic injury population, measurement of walking time and total METmins showed better agreement.

For overall METmins, the moderate concordance between device-based and self-reported measures reported in this study was higher than the correlation previously observed in several other clinical (ρ = 0.43 vs. r = 0.30 and r = 0.37) [9,13] and healthy populations (ρ = 0.43 vs. r = 0.34 and r = 0.09) [35,36] that have also used the IPAQ. The agreement between the IPAQ and activity monitors for measurement of METmins was higher than in previous validation studies which, along with the fact that this measure accounts for all activity (MPA, VPA, walking), highlights the relevance of this measure in the orthopaedic trauma population. There is also the potential to include measurement of LPA either as part of the METmins calculation or as a stand-alone variable when assessing this population’s activity profile.

For sitting time, our study and previous studies in clinical and healthy populations have shown slight to fair correlation between self-reported using the IPAQ and device-based measures (ρ = 0.38 vs. r = 0.36 and r = 0.15) [9,38] and an underestimation of device-measured sitting time [38]. Despite this, sitting remains an important outcome to measure in people with an orthopaedic injury. People with upper limb injury under-estimated their total daily sitting time to a greater extent than lower limb injury patients, which may be due to a perception that recovery from a lower limb injury requires the majority of the day to be spent sitting as compared to upper limb injury patients who have fewer mobility restrictions. Given recent reports of people with an orthopaedic injury spending an average of 11.07 h/day sitting [39], these findings suggest that targeted education interventions are required that encourage physically capable orthopaedic patients to limit their sedentary time.

There are a number of potential clinical implications to our findings. We have documented quite striking differences between the self-reported measures of walking, total physical activity and sitting time, as follows: Walking time: 2.34 h/week over-estimation (IPAQ > objective); total physical activity (METmins): 767 METmins/week over-estimation (IPAQ > objective); and, sitting time: 2.26 h/day under-estimation (objective > IPAQ). For clinicians and other healthcare providers, it will be important to be wary of taking patient self-reports at face value, given these quite large overestimations of physical activity and underestimation of time spent sitting. In most circumstances, given these under- and overestimations, it would likely be helpful to advise patients to do their best to increase activity levels and decreased time spent sitting, within what would be clinically prudent and safe constraints.

Objective activity monitoring may not be feasible in many settings such as when assessing pre-injury physical activity, the acute hospital setting and in disadvantaged communities where the cost of accelerometers is prohibitive. The use of a validated, easy-to-administer questionnaire may provide physicians with a method for assessing physical activity in a patient in whom this measurement may otherwise be overlooked. Assessing physical activity levels in orthopaedic patients may not only aid in recovery, but also has the potential to prompt an intervention that will reduce the patient’s risk of developing associated chronic health conditions, and ultimately the associated health and economic burden.

A limitation of this study is that the activPAL and ActiGraph have not been previously validated in orthopaedic injury patients or under non-weightbearing conditions, despite these being the two most commonly used activity monitors in the current research [16,17]. However, activPAL has been tested and shown to be appropriate in slow walking conditions and in the presence of gait aids [18]. Participants were required to remember activity they performed up to 10 days prior, and thus recall bias may have limited the study findings. As the IPAQ was created prior to recent changes in physical activity guidelines [40] and for assessing physical activity at a population level, it only allows for activity bouts longer than 10 min to be recorded [20]. However, patients in the unique target population sampled in the current study were not expected to be performing long bouts of physical activity. Consequently, a large number of zero values were reported for each of the moderate, vigorous and walking activity categories. Removing this requirement as well as focusing on light physical activity would ensure that the IPAQ more specifically addresses the activities performed by this population. This was the first study, in adults, to determine the agreement between the IPAQ and activity monitors in both an acute and an orthopaedic injury population, and agreement was measured during a single time period. Valid measurement is the cornerstone of treatment prescription and research conclusions. Ensuring the validity of self-report methods, such as the IPAQ, is also of particular importance, as they are the only methods for assessing physical activity and sedentary behaviour both before and after injury. This research may, therefore, help both clinicians and researchers to ensure their actions are reflective of real-world practice.

## 5. Conclusions

This study showed that the self-reported Short-Form IPAQ can significantly overestimate device-measured physical activity and underestimate sitting time in people with orthopaedic injury. The physical activity categories defined by the IPAQ are broad and non-specific, particularly for low levels of physical activity. Based on our findings, focusing measurement on domains of physical activity and sedentary behaviour that are more appropriate to orthopaedic injury patients (i.e., walking, LPA and sitting) may be required. This may involve either modification of the IPAQ, or the use of a more tailored assessment tool such as the ‘CHAMPS Physical Activity Questionnaire for Older Adults’ that is designed for assessing physical activity in a less active patient group.

## Figures and Tables

**Table 1:** Demographic characteristics of study sample (*n* = 46).

		Mean (SD) or Frequency (%)
Age (years)		40.5 (14.4)
Height (cm)		174 (9.2)
Weight (kg)		77.0 (16.0)
Body Mass Index (kg/m^2^)		25.5 (4.7)
Female		17 (37%)
Injury Location	Upper limb (UL)	22 (48%)
	Lower limb (LL)	24 (52%)
Weight-bearing status (on injured side)	Non-weight bearing	20 (44%)
	Touch/Partial weight bearing	3 (7%)
	Full weight bearing	1 (2%)
	N/A (Upper limb injury)	22 (48%)

**Table 2:** Physical activity and sitting time data from the International Physical Activity Questionnaire– Short Form (IPAQ) and activity monitors by variable and injury location.

		Self-Reported (IPAQ)		Device-Measured	
		Median (IQR)	Participants with Zero Value, *n* (%)	Median (IQR)	Participants with Zero Value, *n* (%)
Walking (h/week)	All participants	2.25 (0.17, 7.00)	8 (20)	0.00 (0.00, 0.94)	28 (70)
	UL injury	3.50 (1.25, 7.00)	3 (16)	0.33 (0.00, 4.80)	9 (47)
	LL injury	0.75 (0.17, 4.00)	5 (24)	0.00 (0.00, 0.00)	19 (91)
MPA (h/week)		0.0 (0.0, 0.0)	37 (82)	0.0 (0.0, 0.4)	33 (73)
VPA (h/week)		0.0 (0.0, 0.0)	40 (89)	0.0 (0.0, 0.0)	44 (98)
METmins (total/week)	All participants	550 (140, 1725)	5 (13)	0 (0, 487)	27 (68)
	UL injury	693 (396, 2376)	2 (11)	306 (0, 1542)	8 (42)
	LL injury	264 (66, 924)	3 (14)	0 (0, 0)	19 (91)
Sitting (hr/day)	All participants	8.00 (5.00, 11.00)	0	10.59 (9.81, 12.05)	0
	UL injury	6.00 (4.50, 8.00)	0	10.04 (8.71, 10.53)	0
	LL injury	10.50 (6.00, 12.50)	0	11.73 (10.68, 12.87)	0

Moderate-intensity Physical Activity (MPA), Vigorous-intensity Physical Activity (VPA), Metabolic Equivalent minutes (METmins), upper limb (UL), lower limb (LL).

**Table 3:** Average mean difference (over/under-estimation) between the International Physical Activity Questionnaire–Short Form and device measured physical activity and sitting time.

		Average Mean Difference between Measurement Methods (IPAQ-Device Derived)	Limits of Agreement
Walking	All participants	2.34 h/week	±7.33 h/week
	Upper limb	2.00 h/week	±8.56 h/week
	Lower limb	2.67 h/week	±1.54 h/week
METmins	All participants	767 METmins/week	±1659 METmins/week
	Upper limb	721 METmins/week	±2089 METmins/week
	Lower limb	760 METmins/week	±410.5 METmins/week
Sitting	All participants	−2.26 h/day	±3.87 h/day
	Upper limb	−3.41 h/day	±3.34 h/day
	Lower limb	−1.45 h/day	±4.48 h/day

International Physical Activity Questionnaire (IPAQ), Metabolic Equivalent minutes (METmins).

**Table 4:** Concordance between the International Physical Activity Questionnaire–Short Form and device-based measurement methods for assessing physical activity and sitting time.

		ρ	95% CI	*p*-Value
Walking	All participants	0.34	0.10, 0.58	0.01
	Upper limb	0.43	0.06, 0.79	0.02
	Lower limb	−0.02	−0.11, 0.07	0.65
Moderate PA	All participants	0.21	0.06, 0.36	0.01
Vigorous PA	All participants	0.03	0.01, 0.04	<0.001
METmins	All participants	0.43	0.25, 0.62	<0.001
	Upper limb	0.54	0.28, 0.80	<0.001
	Lower limb	−0.01	−0.09, 0.06	0.71
Sitting	All participants	0.38	0.23, 0.54	<0.001
	Upper limb	0.19	0.02, 0.35	0.03
	Lower limb	0.33	0.08, 0.58	0.01

Physical Activity (PA), Metabolic Equivalent minutes (METmins). ρ: Lin’s Concordance Correlation Coefficient.

**Table 5:** Kappa 2 × 2 contingency table (Overall physical activity categories) and Weighted Kappa statistics.

	IPAQ Low	IPAQ Medium	IPAQ High
Device-derived Low	23	8	2
Device-derived Medium	1	2	2
Device-derived High	0	1	1
	ĸ	95% CI	*p*-value
All Participants	0.33	0.10, 0.57	<0.01

IPAQ (International Physical Activity Questionnaire).

## Supplementary Materials

The following are available online at https://www.mdpi.com/1660-4601/17/17/6139/s1, Figure S1: Schematic of study methods. IPAQ (International Physical Activity Questionnaire).
