# Supplementary material for: Agreement between the International Physical Activity Questionnaire and Accelerometry in Adults with Orthopaedic Injury
Source: Int J Environ Res Public Health. 2020 Aug 24;17(17):6139. doi: 10.3390/ijerph17176139 (PMC7504024; doi:10.3390/ijerph17176139)
Supplement: Supplementary file 1 [file ijerph-17-06139-s001.pdf]

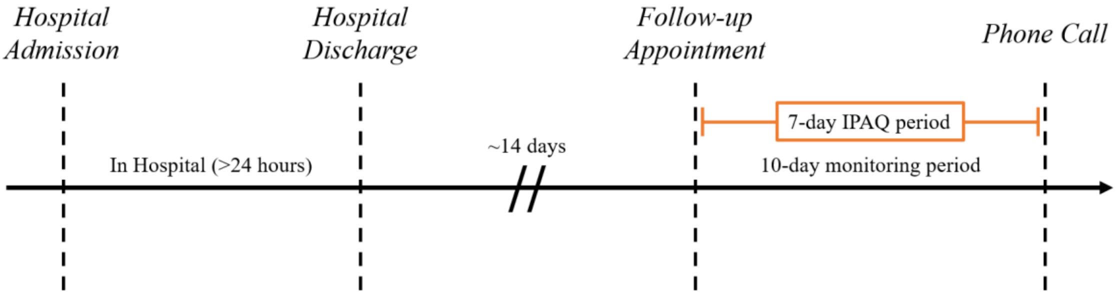

Supplementary Figure 1: Schematic of study methods. IPAQ (International Physical Activity Questionnaire).
